# Commentary: Fear of Massive Deportations in the United States: Social Implications on Deprived Pediatric Communities

**DOI:** 10.3389/fped.2018.00009

**Published:** 2018-01-25

**Authors:** Chris Fradkin

**Affiliations:** ^1^Instituto de Psicologia, Universidade Federal do Rio de Janeiro, Rio de Janeiro, Brazil; ^2^Departamento de Psicologia, Pontifícia Universidade Católica, Rio de Janeiro, Brazil; ^3^Psychological Sciences, University of California, Merced, Merced, CA, United States

**Keywords:** deportation, children, undocumented, Immigration and Customs Enforcement, Deferred Action for Childhood Arrivals

The issue at hand is deportation. The setting is the United States. The subjects are the children of undocumented immigrants, who for the most part come from Mexico or Central America. In the opinion piece by Leiner and colleagues (1), the authors list the consequences of “fear of deportation,” which affect many of these children and their families. These include parents not driving their children to school for fear of deportation; families not reporting domestic abuse for fear of deportation; families not seeking urgent or preventative health care for fear of deportation; reduced opportunities for food and family housing for fear of deportation; and missed opportunities for planning for the future (1). The authors also list the consequences of stress-related illness, including higher levels of: anxiety- and trauma-related illnesses, depressive-related illnesses, family instability, all directly tied to “fear of deportation” (1).

In the first year of one of the most nationalist administrations in American history, this piece reminds us that the well-being of a nation’s children should subsume its political agenda. While the authors build a compelling case for why “fear of deportation” merits our attention, further details could drive their message home. These details are from findings of recent research studies, which support the authors’ well-intentioned piece.

The first finding pertains to the protection that the “Deferred Action for Childhood Arrivals” (DACA) affords children of DACA-enrolled mothers—mothers whose enrollment exempts them from deportation. In a study of adjustment and anxiety disorder among children (*N* = 8,610) born in Oregon, and thereby US citizens, Hainmueller and colleagues (2) saw that children of DACA-eligible mothers had more than 50% fewer diagnoses of adjustment and anxiety disorder (4.3%) than children of non-DACA-eligible mothers (7.9%). This is evidence of the intergenerational protection that DACA provides to children of non-documented mothers.

In an examination of the impact Immigration and Customs Enforcement (ICE) raids have on adverse health outcomes, Novak and colleagues (3) examined birth weight among infants (*N* = 52,344) born prior to and after a major ICE raid (Postville, IA, USA, May 12, 2008; 297 undocumented deported). The authors found that infants born of Latina mothers after the ICE raid had a 24% higher likelihood of low birth weight (LBW) than those infants born prior to the raid, risk ratio = 1.24 (95% CI: 0.98, 1.57). Among non-Latina White mothers, no difference was observed. Studies indicate that infants with LBW have a higher likelihood of diabetes (4, 5), heart disease (6), high blood pressure (4, 7), and obesity (4) later in their lives, than infants of healthy birth weight.

Another examination of immigration raids revealed the stress and trauma toll upon the children. In this study, Rojas-Flores and colleagues (8) examined internalizing, externalizing, and stress-related disorders among a sample of Latino US-born children (*N* = 91). In examining differences based on parental immigration status, the authors found higher levels of posttraumatic stress disorder symptoms among those children of detained or deported parents than among those children of parents not detained or yet deported, *F* = 9.70, *p* < 0.001. These are further findings of the health threats posed to children by today’s aggressive enforcement of immigration laws.

These studies offer evidence of health threats posed to children—US citizens by birth—by the heightened use of immigration raids. This increased frequency is diametrically opposed to the health and welfare of our children.

In their closing remarks, Leiner and colleagues (1) remind us of our need to provide care and protection for our children. This is a priority above and beyond the pale of politics. They say, “the force resulting from the fear of massive deportations will not only affect non-citizen families but will also affect every person in our country” [(1), p. 3]. Nelson Mandela frames this crisis in his time-tested words: “There can be no keener revelation of a society’s soul than the way in which it treats its children” (9), words as apropos today as when he wrote them.

To their credit, Leiner and colleagues (1) have kept the dialog alive. It is a crucial dialog for our survival. As a country, as a species, we must protect our children. To our credit, some steps have now been taken. For one, The Presidents’ Alliance on Higher Education and Immigration (est. 2017), a group of high-level university and college administrators, supports undocumented students on their campuses (10). We see organizations like the East Bay Community Law Center, the UC Immigrant Legal Services Center, and the Immigration Clinic at the University of Texas School of Law, providing free legal aid to undocumented students and their families.

Until the administration pulls back in its stance toward deportation, protection for our children is at risk. But there will still be those like Brenda Eskenazi, of the University of California, who inform the Mexican workers that she studies (a portion of whom do not have legal status) of their rights should ICE come to their door (11). In the meantime, with the help of Dr. Eskenazi, and with others, maybe we can minimize the damage that is done.

## Author Contributions

The author confirms being the sole contributor of this work and approved it for publication.

## Conflict of Interest Statement

The author declares that the research was conducted in the absence of any commercial or financial relationships that could be construed as a potential conflict of interest.
